# Eye Movements in Parkinson’s Disease and Inherited Parkinsonian Syndromes

**DOI:** 10.3389/fneur.2017.00592

**Published:** 2017-11-09

**Authors:** Elena Pretegiani, Lance M. Optican

**Affiliations:** ^1^Laboratory of Sensorimotor Research, National Eye Institute, NIH, Bethesda, MD, United States

**Keywords:** saccades, basal ganglia, α-synuclein, PARK, manganese, Gaucher disease, brain iron accumulation, parkinsonism

## Abstract

Despite extensive research, the functions of the basal ganglia (BG) in movement control have not been fully understood. Eye movements, particularly saccades, are convenient indicators of BG function. Here, we review the main oculomotor findings reported in Parkinson’s disease (PD) and genetic parkinsonian syndromes. PD is a progressive, neurodegenerative disorder caused by dopaminergic cell loss within the substantia nigra pars compacta, resulting in depletion of striatal dopamine and subsequent increased inhibitory BG output from the internal globus pallidus and the substantia nigra pars reticulata. Eye movement abnormalities are common in PD: anomalies are more evident in voluntary than reflexive saccades in the initial stages, but visually guided saccades may also be involved at later stages. Saccadic hypometria (including abnormally fragmented saccades), reduced accuracy, and increased latency are among the most prominent deficits. PD patients show also unusually frequent and large square wave jerks and impaired inhibition of reflexive saccades when voluntary mirror saccades are required. Poor convergence ability and altered pursuit are common. Inherited parkinsonisms are a heterogeneous group of rare syndromes due to gene mutations causing symptoms resembling those of PD. Eye movement characteristics of some parkinsonisms have been studied. While sharing some PD features, each syndrome has a distinctive profile that could contribute to better define the clinical phenotype of parkinsonian disorders. Moreover, because the pathogenesis and the underlying neural circuit failure of inherited parkinsonisms are often well defined, they might offer a better prospect than idiopathic PD to understand the BG function.

## Introduction

The basal ganglia (BG) are subcortical nuclei located at the base of the forebrain and extensively connected directly and indirectly with all cortical and subcortical structures. The BG promote the initiation of goal-directed movement by removing sustained inhibition of the desired movement and suppressing unwanted movements. Despite considerable advancements in understanding the BG anatomy and function, their complex role in modulating motor behavior, remains far from fully elucidated. The saccadic system offers unique advantages in studying the BG because the neural circuits underlying it are relatively well understood and their functional corticobasal loops are likely similar to those involved in regulating other movements (1). Moreover, saccades can be easily and accurately measured.

Eye movements, and particularly the saccadic system, allow to test a distributed network involving cortical (mainly frontal and parietal) and subcortical (BG, midbrain, brain stem, thalamus, and cerebellum) structures. Other than clinically assessed, eye movements can be quantified through electro-oculography, scleral search coil system, and video-oculography. While electrooculography is the only system allowing recording of eye movement with closed eyes, and search coil contact lenses provide the best temporal and spatial resolution, video-oculography is the most used technique given its non-invasiveness (2).

The saccadic system is usually explored by testing reflexive saccades (pro-saccades) toward a visual stimulus that suddenly appears simultaneously, after (gap paradigm), or overlapping (overlap paradigm), the offset of a fixation point exposure. With respect to the simultaneous condition, latency is usually shorter with a gap and longer with an overlap (3). The antisaccade paradigm, in which the saccade is directed to the opposite direction than the stimulus, is used to test voluntary eye movements and inhibition of reflexive movements. The dorsolateral prefrontal cortex is supposed to be involved in the suppression of the unwanted reflexive movement and, with the posterior-parietal cortex, in the generation of the correct mirror movement, while the frontal eye field (FEF) is associated with antisaccade latency (4). Fixation, memory guided saccades toward previously briefly exposed stimuli, and smooth pursuits are also commonly applied to the evaluation of BG function.

Specific eye movement abnormalities follow BG dysfunction (5, 6). Therefore, eye movements are often analyzed to differentiate Parkinson’s disease (PD) from other parkinsonian syndromes. Indeed, ocular motor abnormalities of idiopathic neurodegenerative parkinsonisms such as progressive supranuclear palsy, multisystem atrophy, corticobasal syndrome, and dementia with Lewy bodies, have been extensively studied and are well known by clinicians (2, 6). Yet, eye movements in genetic parkinsonisms are seldom investigated. Nonetheless, eye movement features might support the differential diagnosis of genetic syndromes. Moreover, inherited diseases with known pathogenesis and neurodegenerative progression might offer a better prospect than idiopathic PD to delineate the neural circuits underlying specific failures of the BG.

Here, we report the main findings of oculomotor studies in PD and genetic parkinsonian syndromes (Table 1).

**Table 1:** Main saccadic features in PD and genetic parkinsonisms.

	PD	PARK1	PARK2	PARK6	PARK9	HMNDYT1	NBI	Gaucher disease
**Horizontal saccades**								

Latency	Norm/↑	↑	Norm	↑	Norm/↑	↑	NA	↑
Gain	↓	Norm	↓	Norm	↓	Norm	Norm	↓
Precision	↓	Norm	NA	NA	↓	↓	NA	↓
Velocity	Norm	Norm	Norm	Norm	↓	Norm	Norm	↓

**Vertical saccades**								

Latency	Norm/↑	↑	Norm	NA	↑	↑	NA	Norm
Gain	↓	Norm/↓	↓	NA	↓	Norm	↓	↓
Precision	↓	Norm/↓	NA	NA	↓	↓	↓	↓
Velocity	Norm	Norm	Norm	NA	↓	Norm	↓	↓
Multistep frequency	↑	↑	NA	↑	↑	↑	NA	NA

**Antisaccades**								

Latency	↑	↑	Norm	Norm	↑	↑	NA	NA
Errors	↑	↑	↑	Norm	↑	↑	NA	NA
Corrections	NA	Norm	NA	NA	↓	Norm	NA	NA

*PD, Parkinson’s disease; PARK, Parkinson’s disease-related locus; HMNDYT1, hypermanganesemia with dystonia, polycythemia, and cirrhosis; NBI, neurodegeneration with brain iron accumulation*.

### Parkinson’s Disease

Parkinson’s disease is a progressive, neurodegenerative disorder. Classic clinical manifestations are tremor at rest, muscular rigidity, akinesia (or bradykinesia), and postural instability (7, 8). Included in the typical features of PD are flexed posture and freezing of gait. Non-motor symptoms such as cognitive impairment, apathy, depression, anosmia, dysautonomia, and sleep disorder are also common.

Parkinson’s disease motor manifestations are caused by dopaminergic cell loss within the substantia nigra pars compacta (SNc), resulting in dysfunction of the BG. A cardinal neuro-pathological feature is the development of intracytoplasmic aggregates of α-synuclein, termed Lewy bodies. Because the dopaminergic neurons in SNc project to the striatum (caudate and putamen), SNc cell loss results in depletion of striatal dopamine (9). PD motor symptoms are recognized when 60% of SNc cells are lost, corresponding to 80% depletion of striatal dopamine.

**Figure 1:**
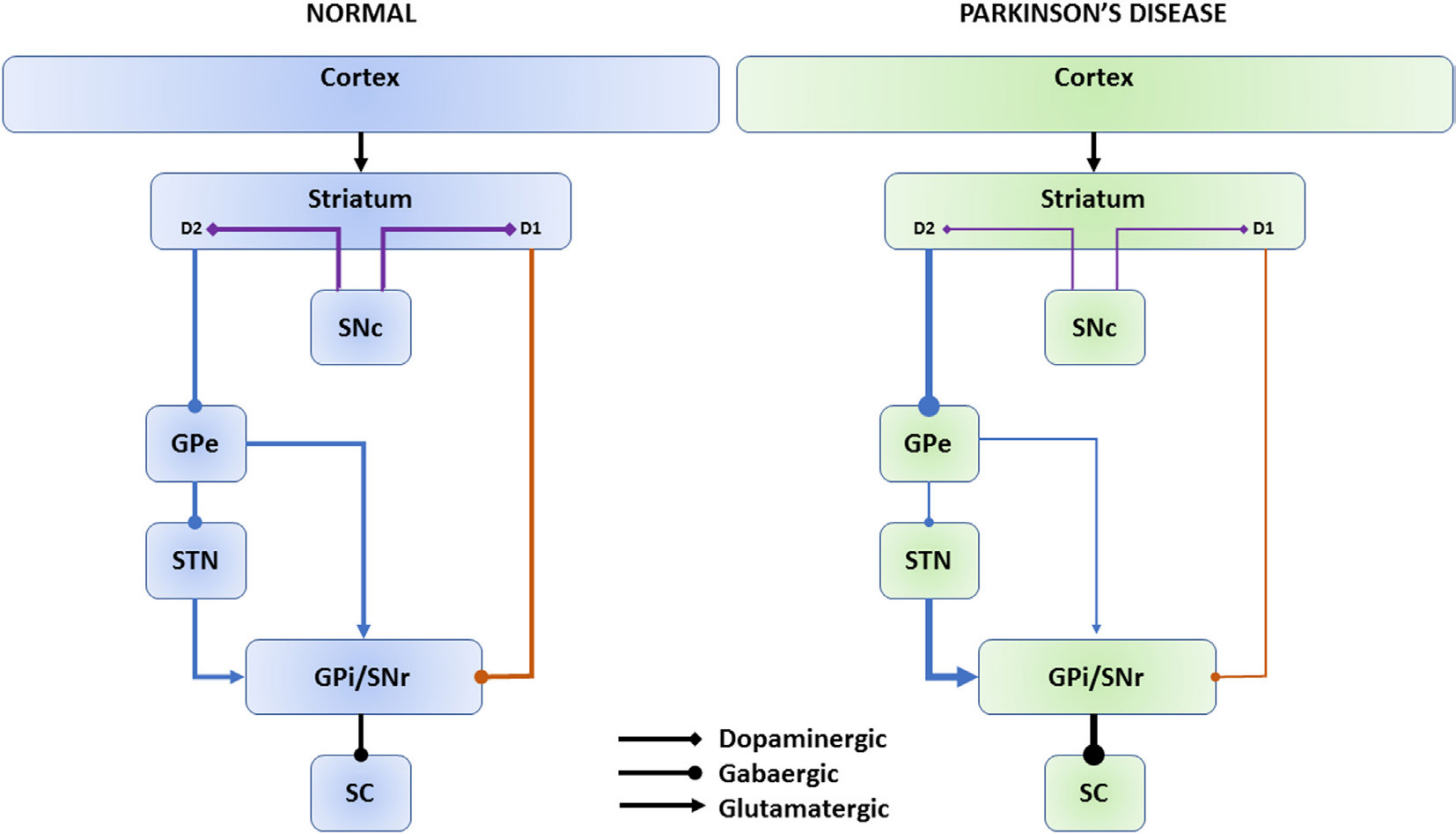
Direct and indirect basal ganglia pathways. The cerebral cortex sends input to the striatum. Dopaminergic projections from the SNc (violet connectors) target striatal neurons expressing D1 or D2 receptors. Direct pathway (orange connectors): D1 neurons send direct inhibitory projections to the SNr/GPi. Indirect pathway (blue connectors): D2 neurons connect indirectly to the GPi/SNr through the GPe and STN. The SNr inhibits the SC. In Parkinson’s disease, dopaminergic depletion leads to reduced inhibitory direct pathway output (thin lines) and increased excitatory indirect pathway output (thick lines) onto the GPi/SNr and, consequently, increased SNr inhibition onto the SC as net effect. SNc, substantia nigra pars compacta; GPe, external globus pallidus; STN, subthalamic nucleus; GPi, internal globus pallidus; SNr, substantia nigra pars reticulata; SC, superior colliculus.

While partly challenged by more recent findings, the classical model depicts two parallel pathways connecting the BG nuclei (Figure 1) (10). The striatum receives input from the FEF, supplementary eye field, DLPFC, and the parietal eye field (PEF). In the direct pathway, dopaminergic projections from the SNc target striatal neurons expressing D1 receptors; D1 neurons send direct inhibitory projections to the BG output nuclei: the internal globus pallidus (GPi) and the substantia nigra pars reticulata (SNr). In the indirect pathway, D2 expressing neurons receive projections from the SNc and connect indirectly to the GPi/SNr through the external globus pallidus (GPe) and the subthalamic nucleus (STN) (11). The SNr inhibits the superior colliculus (SC). The SC is a crucial structure for both voluntary and reflexive saccades. The SC, indeed, integrates visual, somato-sensory, and auditory stimuli in a spatial map and produces a motor saccade command that is sent to the brain stem saccade generators. The cortical-BG-SC pathway is supposed to be particularly important in selecting the most appropriate or most rewarding movement when multiple internal and/or external inputs compete to orient the body, or the eyes, to different locations (12, 13).

In PD, dopaminergic depletion leads to reduced inhibitory direct pathway output and increased excitatory indirect pathway output onto the GPi/SNr (8, 14). The hyperactivated SNr induces excessive inhibition of the SC, which is considered to be responsible for the typical eye movement abnormalities in PD (15).

However, voluntary saccade generation involves a cortex-BG-SC pathway, whereas reflexive saccades can be generated by direct projections from the parietal cortex onto saccade-related neurons in the intermediate layer of the SC (16). This processing is supposed to largely bypass the BG circuit and it is considered the reason why reflexive saccades are mostly preserved in PD, particularly at early stages of the disease, while voluntary saccades are more severely affected (15, 17). According to this picture, a recent fMRI study, while failing to find differences in saccadic metrics between PD patients and controls, found that PD showed left frontal underactivation during horizontal prosaccades and right parietal overactivation during horizontal and vertical prosaccades and horizontal antisaccades (18).

One of the most prominent features of eye movement abnormality in PD is saccade hypometria (15, 19). As expected, hypometria is more severe in voluntary saccades, particularly in memory-guided saccades, where a subject is required to make a saccade to a remembered target location. Reflexive saccades, usually preserved in the initial stages of PD, can become hypometric in later stages (2). Reflexive saccade hypometria is thought to result solely from excessive SC inhibition, compared to hypometric voluntary saccades that are supposed to be caused by both increased SC inhibition and reduced pre-oculomotor drive due to dysfunctional frontal cortex-BG-SC circuit (20). An alternative explanation for hypometria in PD involves a dysfunction of the cerebellum which is hyperactivated in PD (21). However, a fundamentally preserved saccadic adaptive ability in PD suggests normal cerebellar function, at least during the early stage of the disease (22).

Abnormally fragmented saccades, called multistep or staircase saccades, have been described in PD. These movements, where the target is reached by several hypometric saccades, are observed also in normal subjects (23), but they are more frequent when PD patients execute memory-guided or self-paced saccades (24). Their exact mechanism is still not understood. Gaze fragmentation is supposed to reflect an inappropriate inhibition of the saccade generator (25, 26). Therefore, multistep saccades in PD might be an expression of saccadic hypometria or improper reactivation of omnipause neurons due to SC dysfunction (27), but they could also just reflect a general facilitation in the execution of small saccades (20).

Latency of voluntary saccades is usually delayed in PD, indicating difficulty in initiating volitional eye movements. Latency of reflexive saccades can be spared during the early stages of the disease, when PD patients may produce saccades even faster than normal (express saccades), particularly for small target eccentricities. This facilitation disappears at advanced stages when latency of reflexive saccades also increases, particularly for large target eccentricities (20).

Parkinson’s disease patients show also impairment of inhibition of reflexive saccades to a visual cue when a voluntary mirror saccade is required (so-called antisaccades) (28). Dysfunction of the suppression of unwanted saccades in the DLPFC following dopaminergic depletion in the prefrontal cortex, “leaky” suppression of the SC from the BG, and cognitive impairment have all been associated with impaired saccadic inhibition and “hyper-reflexivity” in PD (4, 15, 29). Antisaccades can reveal deficits of executive functions even in early stages of PD (30). Recently, antisaccade errors have been related to freezing of gait, and increased antisaccade latency in PD has been correlated with impaired postural control (31, 32).

Square wave jerks are saccadic intrusions (usually 0.5–5°) that move the eye from and back to the fixation point with an intersaccadic interval of about 200 ms. Abnormally frequent and large square wave jerks in PD patients have been ascribed to compensatory increased activity in the FEF (19, 26). PD patients show other abnormal eye movements such as poor convergence ability and altered pursuit (33). Pursuit is often saccadic in PD patients who can also show increased pursuit latency and reduced gain and impaired preparation and execution of cue-dependent memory-based smooth-pursuits (34, 35).

A long debate has characterized the finding of ocular tremor in PD patients (36–38). Indeed, while some researchers support the presence of pervasive ocular tremor in PD (39, 40), others consider it the simple consequence of the vestibulo-ocular reflex induced by head movement (41, 42).

Eye movement abnormalities are supposed to impair some behaviors of PD patients. Deficient generation of voluntary saccades, for example, might explain the visual search pattern of PD: patients scan smaller areas than normal with fewer, hypometric saccades, which could lead to a mild degree of visuospatial neglect in PD patients (43). Facilitation of small saccades might underlie reading difficulties (44). Impairment in the generation of voluntary saccades can also affect stability and walking (6).

While impaired oculomotor performance is attributed to dopamine depletion, studies on the effect of dopaminergic treatment on eye movement have given inconsistent results (45, 46).

Finally, PD patients show several oculo-visual dysfunctions (i.e., hallucinations and impairment of visual acuity, color and contrast sensitivity, motion perception, stereopsis), but whether and how they impact eye movement is difficult to establish (47, 48).

### Parkinson Disease 1 (*PARK1*)

Parkinson disease 1 (*PARK1*, OMIM number #168601) is the first genetically identified parkinsonism. Its associated autosomal dominant mutation in gene *SNCA* was initially isolated in Italian families (49). *SNCA* encodes a presynaptic protein, α-synuclein, involved in neuronal plasticity. Intra-neuronal aggregates of α-synuclein are the hallmark of neurodegenerative synucleinopathies, being the major component of Lewy bodies in PD and Lewy body dementia. Tau-inclusions may be also frequent. Neuronal loss is more severe in the brain stem, hippocampus, dorsal motor nucleus of the vagus, SNc, nucleus basalis of Meynert, and locus coeruleus, but it involves also cortical areas (49, 50). The clinical phenotype of patients with mutations in *PARK1* resembles typical sporadic PD, except for earlier onset, rapid progression, and frequent cognitive decline. Eye movement recording from two patients with mutated *PARK1* showed increased latency of reflexive and voluntary saccades, more frequent multistep saccades, but normal average gain and precision, and normal saccade velocity and duration; patients made more directional errors at the antisaccade task, but corrected as frequently as normal (51).

### Parkinson Disease 2 (*PARK2*)

Parkinson disease 2 (*PARK2*, #60016) is due to homozygous and compound heterozygous mutations in *Parkin (PRKN)* and it is the most common genetic parkinsonism. Parkin defects interfere with the ubiquitin-mediated proteolytic pathway and cause accumulation of 22-kD glycosylated α-synuclein leading to neurodegeneration with more involvement of the SNc than the locus coeruleus with respect to idiopathic PD (50). Phenotype is similar to that of sporadic PD.

Recording of eye movements in symptomatic patients with P*ARK2* mutations showed hypometric saccades with normal latency, antisaccades with normal latency but increased error rate, and reduced gain of smooth pursuit (52, 53).

### Parkinson Disease 6 (*PARK6*)

Parkinson disease 6 *(PARK6*, #605909) results from mutations in *PINK1* coding a mitochondrial protein (PTEN-induced putative kinase 1), causing increased susceptibility to cellular stress and apoptosis (54). Neurodegeneration affects the SNc, brain stem reticular formation, and nucleus basalis of Meynert and it is associated with cortical and brain stem Lewy bodies (50). Patients present with parkinsonism, gait disturbances, and psychosis. A cohort of *PINK1* mutation carriers showed increased latency of horizontal prosaccades with normal gain and velocity, higher rate of multistep saccades, normal error rate in the antisaccade task; homozygous (but not heterozygous) patients showed also hypometric memory guided saccades (55).

### Parkinson Disease 9 (*PARK9*)

Parkinson disease 9 (*PARK9* or Kufor-Rakeb syndrome, #606693) is a rare autosomal recessive juvenile-onset levodopa-responsive parkinsonism due to mutations in *ATP13A2* (*PARK9*) encoding a lysosomal P-type ATP-ase (56). Mutation of *PARK9* leads to α-synuclein accumulation and increased manganese toxicity (57), causing cortical and subcortical neurodegeneration. Beside progressive parkinsonism patients present also with pyramidal signs, facial-faucial-finger mini-myoclonus, and cognitive decline (58).

Neuroimaging shows reduced dopamine transporter activity and reduced gray matter in the motor cortex, prefrontal cortex, somatosensory association cortex, cingulate, caudate, thalamus, and cerebellum. Accumulation of iron in the BG has been detected in some cases.

Typical eye movement abnormalities are vertical supranuclear gaze palsy, slowing of vertical and horizontal saccades, and saccadic pursuit (58, 59). Eye movement recordings from three patients showed hypometric and slow saccades with worse precision, increased or normal latency of horizontal saccades and increased latency of vertical saccades (51, 60). Target was often reached by multistep saccades. Antisaccades directional error rate was increased and patients never corrected the errors (51).

### Hypermanganesemia with Dystonia, Polycythemia, and Cirrhosis (HMNDYT1)

Hypermanganesemia with dystonia, polycythemia, and cirrhosis (HMNDYT1, #613280) is a parkinsonism due to recessive mutations in *SLC30A10* (61). Loss of function of the encoded manganese transporter leads to a primary metabolic disorder causing hypermanganesemia. Manganese accumulation induces cell toxicity in the liver, bone marrow, and nervous system. Brain MRI T1 hyperintense lesions are present in the caudate and lentiform nuclei, thalamus, corticospinal tract, substantia nigra, posterior pons, and bulbar olives, cerebellum and cerebello-rubro-thalamic pathways.

Eye movement recording from two affected patients showed increased latency of reflexive and voluntary saccades, normal gain and velocity, but worse precision, and increased frequency of multistep saccades. Antisaccade errors were increased, but they were corrected as frequently as normal (51).

Manganese is also a known cause of environmental intoxication upon overexposure in workers and drug abusers (62, 63). In these cases, manganese accumulates in the globus pallidus more than in other brain structures, causing parkinsonism and oculomotor abnormalities. A detailed description of the oculomotor abnormalities is available only for subjects with ephedrone-induced parkinsonism (62). Ephedrone (also called methcathinone or α-methylamino-propiophenone) is a home-made drug obtained by oxidation of ephedrine or pseudoephedrine with potassium permanganate and acetic acid (62). Ephedrone addicts can face manganese intoxication leading to severe, rapidly progressive, non-levodopa responsive parkinsonism and dystonia. Patients showed hypometric and slow horizontal saccades with normal latency, and increased latency of vertical saccades; antisaccades showed normal latency, but increased error rate with normal correction frequency. These findings differ with respect to those shown by subjects with genetic hypermanganesemia, perhaps indicating that the slow manganese accumulation in HMNDYT1 might allow for some adaptation.

### Neurodegeneration with Brain Iron Accumulation

Neurodegeneration with brain iron accumulation is a genetically heterogenous disorder causing progressive accumulation of iron in the BG and other brain regions, most cases being associated with mutations in *PANK2* and *PLA2G6*. Pantothenate kinase-associated neurodegeneration (PKAN, formerly Hallervorden-Spatz syndrome, #234200) is a recessive disease associated with mutations in *PANK2*. Patients present with cognitive dysfunction and extrapyramidal features such as parkinsonism and dystonia. Eye movement abnormalities include hypometric and slow vertical saccades, normal horizontal saccades, saccadic pursuit, impaired vestibulo-ocular reflex suppression, poor convergence, square-wave jerk saccadic intrusions, and abnormal vertical optokinetic reflex (64). Patients with *PLA2G6* mutations (#603604) can present with up gaze palsy, poor convergence, saccadic intrusions, and saccadic pursuit (65).

### Gaucher Disease

Gaucher disease (#230800, #230900, #231000) is the most common autosomal recessive lysosomal storage disorder and it is due to mutations in *GBA* leading to deficit of glucocerebrosidase and intracellular accumulation of glucosylceramide. Typical features are hepatosplenomegaly and pancytopenia. Several patients with Gaucher disease presented with parkinsonism. In these patients, neuronal loss occurred in SNc, hippocampus, and cortex (50). Ocular motor abnormalities have been proposed as an early marker to detect neurological impairment in type 3 Gaucher disease, distinguishing it from the subtype without neurological involvement (type1) (66, 67).

Patients affected by type 3 Gaucher disease showed slow velocity (affecting horizontal more than vertical saccades), saccadic hypometria, and increased horizontal saccade latency (68). Saccadic pursuit and oculomotor apraxia were also reported.

Recently, heterozygous *GBA* mutation carriers have been found to be at higher risk to develop PD and Lewy body dementia (69, 70). The phenotype of these patients can be indistinguishable from that of idiopathic PD.

## Conclusion

Studies of eye movements in inherited parkinsonian syndromes are often limited by small sample sizes and by inconsistent examination techniques and paradigms. Nevertheless, these reports support distinct pictures of eye movement disorders for each genetic syndrome, highlighting some features as possible markers for differential diagnosis and for evaluation of disease extension and progression. Still, some characteristics tend to recur across syndromes and could be more informative about BG functions: saccades with increased latency but normal velocity suggest a role of BG in motor initiation rather than execution, so that bradykinesia in parkinsonism should be interpreted more as a delayed motor onset than a slow movement. Also, increased error rate in the antisaccade task, with often normal frequency of correction, might indicate that BG participate more in selecting than planning the proper movement among competitive movements. Other less frequent features are usually associated with more severe phenotypes, possibly indicating more profound BG damage or degenerative involvement of other brain structures.

More studies, applying the same saccadic paradigms and integrating clinical, genetic, neuroimaging, and neuropathological data, are needed for better picturing the oculomotor features associated with BG dysfunctions in PD and parkinsonisms and their relationship with other motor and non-motor symptoms. Clarifying these aspects might provide clinical/diagnostic indications to guide the evaluation of patients with parkinsonism, including its genetic variants. Also, it will help in understanding the modulatory role of BG in behavior.

## Author Contributions

EP and LO: design of the work, acquisition and interpretation of data for the work, drafting the work, and final approval of the article.

## Conflict of Interest Statement

The authors declare that the research was conducted in the absence of any commercial or financial relationships that could be construed as a potential conflict of interest.
